# Rotating Flow of Magnetite-Water Nanofluid over a Stretching Surface Inspired by Non-Linear Thermal Radiation

**DOI:** 10.1371/journal.pone.0149304

**Published:** 2016-02-19

**Authors:** M. Mustafa, A. Mushtaq, T. Hayat, A. Alsaedi

**Affiliations:** 1 School of Natural Sciences (SNS), National University of Sciences and Technology (NUST), Islamabad, 44000, Pakistan; 2 Research Centre for Modeling and Simulation (RCMS), National University of Sciences and Technology (NUST), Islamabad, 44000, Pakistan; 3 Department of Mathematics, Quaid-I-Azam University 45320, Islamabad, 44000, Pakistan; 4 Nonlinear Analysis and Applied Mathematics (NAAM) Research Group, Department of Mathematics, Faculty of Science, King Abdulaziz University, Jeddah, 21589, Saudi Arabia; North China Electric Power University, CHINA

## Abstract

Present study explores the MHD three-dimensional rotating flow and heat transfer of ferrofluid induced by a radiative surface. The base fluid is considered as water with magnetite-Fe_3_O_4_ nanoparticles. Novel concept of non-linear radiative heat flux is considered which produces a non-linear energy equation in temperature field. Conventional transformations are employed to obtain the self-similar form of the governing differential system. The arising system involves an interesting temperature ratio parameter which is an indicator of small/large temperature differences in the flow. Numerical simulations with high precision are determined by well-known shooting approach. Both uniform stretching and rotation have significant impact on the solutions. The variation in velocity components with the nanoparticle volume fraction is non-monotonic. Local Nusselt number in Fe_3_O_4_–water ferrofluid is larger in comparison to the pure fluid even at low particle concentration.

## Introduction

The study of flow in a rotating frame is motivated in view of its theoretical and practical significance in geophysics and engineering. Prominent geophysical applications include the magma flow in earth’s mantle close to earth crust and flows in geological formations subject to earth rotation. The engineering applications of such flows exist in chemical and food processing industry, centrifugal filtration process, rotating machinery and design of multi-pore distributor in a gas-solid fluidized bed. Pioneering study on the three-dimensional rotating viscous flow induced by a stretching surface was presented by Wang [1]. His problem was governed by an interesting parameter *λ* that signifies the ratio of the rotation to the stretching rate. He constructed series solutions for small values of parameter *λ* by regular perturbation approach. He found that velocity distribution (above the sheet) decreases upon increasing this parameter *λ*. Rajeswari and Nath [2] and Nazar et al. [3] extended the Wang’s work for unsteady case. Their results indicate a smooth transition from initial unsteady flow to final steady-state flow. Homotopy solutions for rotating flow of non-Newtonian second grade fluid were provided by Hayat et al. [4]. They observed that fluid velocity has direct relationship with material parameter of second grade fluid. Zaimi et al. [5] examined the rotating flow of viscoelastic fluid bounded by a stretching surface and concluded that boundary layer thickness is an increasing function of viscoelastic fluid parameter. Rashidi et al. [6] investigated entropy generation in steady MHD flow due to a rotating porous disk in a nanofluid. Sheikholeslami et al. [7] reported numerical results of nanofluid flow and heat transfer in a rotating system with the consideration of magnetic field effects. Mustafa [8] used Cattaneo-Christov heat flux model to investigate the rotating flow of viscoelastic fluid bounded by a stretching surface.

Cooling capabilities of heat transfer equipment have been constrained because of the low thermal conductivity of conventional coolants including water, oil and ethylene glycol. It is well known that thermal conductivity of metals is very high as compared to that of liquids. Thus one of effective approaches to enhance heat transfer performance is via dispersing tiny metallic particle of nanometer dimensions in the liquids. Choi [9] was the first to introduce the terminology of nanofluids. Sheikholeslami and Ganji [10] considered the MHD flow and heat transfer inside a semi annulus enclosure having sinusoidal hot wall. They used Maxwell models to estimate the effective thermal conductivity and effective electrical conductivity of the nanofluids. Turkyilmazoglu [11] examined the flow of five different types of water based nanofluids due to rotating disk. He employed a spectral Chebyshev collocation method to present numerical solutions of the developed nonlinear problem. The study of nanofluid convective heat transfer has been a popular research topic for the last several years [12–21]. An electrically conducting nanofluid subject to magnetic field, known as ferrofluid, has been found pretty useful in several applications. Ferrofluid comprises of iron based nanoparticles such as magnetite, hematite, cobalt ferrite or some other compounds containing iron. Such iron-based nanoparticles can be used for efficient drug delivery by guiding the particles via external magnets [22, 23]. Particularly, magnetic nanoparticles are prominent in hyperthermia, magnetic cell separation, cancer tumor treatment (radiotherapy and chemotherapy) and contrast enhancement in magnetic resonance imaging (MRI). Jue [24] used semi implicit finite element method in order to simulate magnetic gradient and thermal buoyancy induced cavity ferrofluid flow. Influence of magnetic field dependent viscosity on the horizontal layer of ferrofluid was addressed by Nanjundappa et al. [25]. Natural convection flow of Fe_3_O_4_-water nanofluid through a rectangular enclosure containing an oval-shaped heat source was considered by Moraveji and Hejazian [26]. MHD flow and heat transfer characteristics in a rotating cylinder was examined by Selimefendigil and Oztop [27]. They observed that cylinder rotation strongly influence the heat transfer rate at low Reynolds number. Ellahi [28] presented the analytical solutions for MHD flow of non-Newtonian nanofluid in a pipe. He also considered temperature dependent viscosity in his analysis. Bahirael and Hangi [29] investigated the heat transfer performance of Mn-Zn ferrite-water ferrofluid in a counter-flow double-pipe heat exchanger under the effect of magnetic field. In another paper, Bahirael et al. [30] examined the flow of Mn-Zn ferrite-water ferrofluid through an annulus under the influence of non-uniform magnetic field. Recently, effect of magnetic field on nanofluid flow and heat transfer has been investigated by several authors [31–40].

To our knowledge, the rotating flow of ferrofluids developed by stretching surface has never been explored. Thus present work is undertaken to fill such a void. Interesting aspect of nonlinear radiative heat transfer is also accounted. This allows us to analyze the problem for both large and small temperature differences between the stretching surface and ambient fluid. Some studies pertaining to the non-linear radiative heat transfer have appeared in the recent past (see [41–46] for details). The governing non-linear differential system has been dealt with shooting technique combined with Newton method. Emphasis has been given to the role of embedding parameters on the velocity and temperature functions. In shooting method, the boundary conditions are assumed as multivariate functions of the initial conditions at a particular point. It acquires advantage of faster convergence and simple implementation of the methods for initial value problems such as fourth-fifth-order Runge-Kutta (RK45) method.

## Mathematical Model

**Fig 1:**
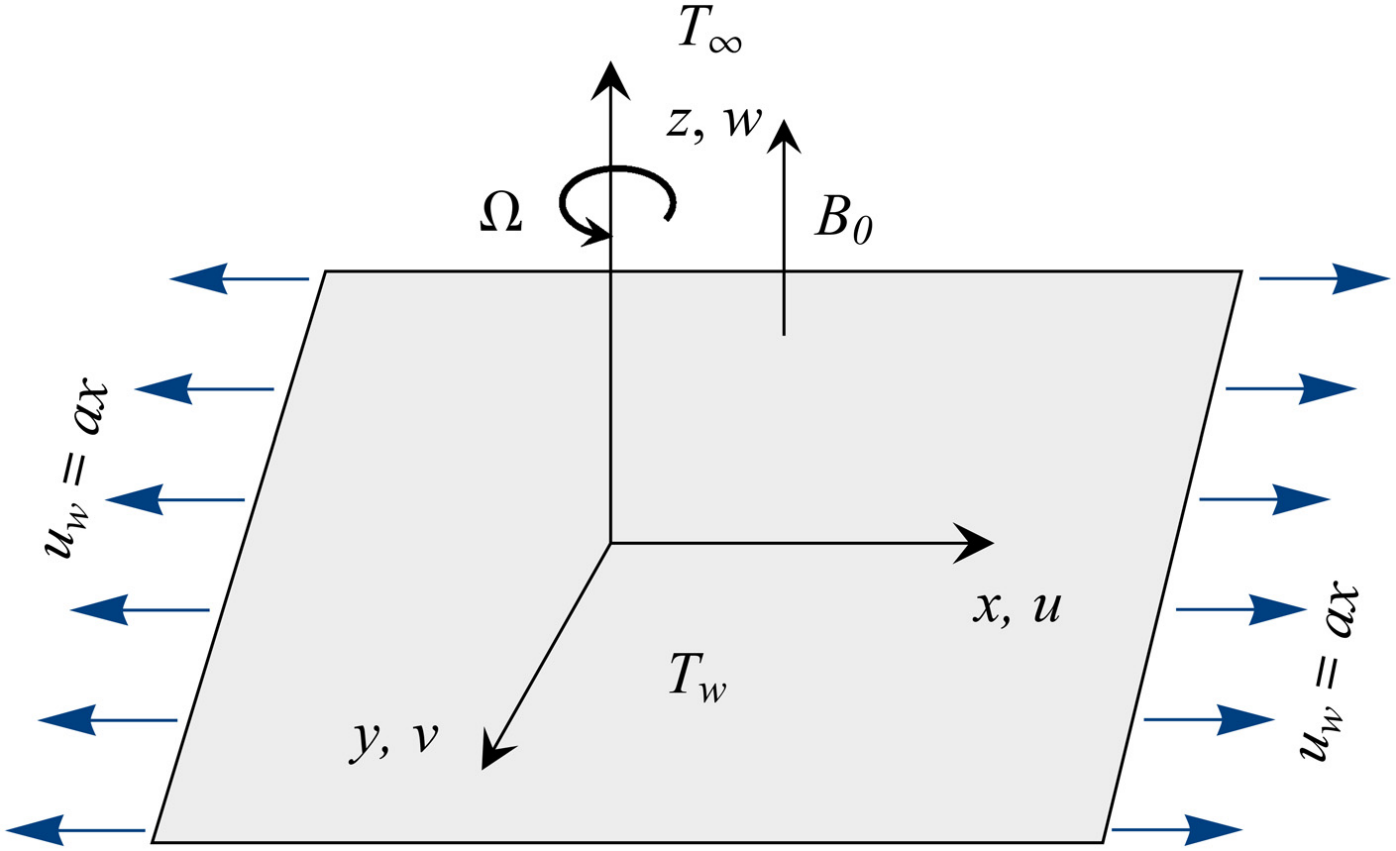
Geometry of the problem and coordinate system.

Consider the rotating flow of Fe_3_O_4_-water ferrofluid caused by a radiative surface coincident with the *xy*− plane. The surface is subjected to uniform stretch in the *x*− direction with rate *a*. The sheet is maintained at a constant temperature *T*_*w*_ whereas *T*_∞_ denotes the temperature outside the thermal boundary layer (see Fig 1). Following the famous Tiwari and Das model [47], the equations embodying the conservation of mass, momentum and energy are expressed as below:

$$\frac{\partial u}{\partial x}+\frac{\partial v}{\partial y}+\frac{\partial w}{\partial z}=0,$$ (1)

$$\rho_{nf}\left(u\frac{\partial u}{\partial x}+v\frac{\partial u}{\partial y}+w\frac{\partial u}{\partial z}-2\Omega v\right)=\mu_{nf}\left(\frac{\partial^{2}u}{\partial z^{2}}\right)-\sigma_{nf}B_{0}{}^{2}u,$$ (2)

$$\rho_{nf}\left(u\frac{\partial v}{\partial x}+v\frac{\partial v}{\partial y}+w\frac{\partial v}{\partial z}+2\Omega u\right)=\mu_{nf}\left(\frac{\partial^{2}v}{\partial z^{2}}\right)-\sigma_{nf}B_{0}{}^{2}v,$$ (3)

$$u\frac{\partial T}{\partial x}+v\frac{\partial T}{\partial y}+w\frac{\partial T}{\partial z}=\alpha_{nf}\left(\frac{\partial^{2}T}{\partial z^{2}}\right)-\frac{1}{{\left(\rho c_{p}\right)}_{nf}}\frac{\partial q_{r}}{\partial z},$$ (4)

with the following boundary conditions

$$\begin{array}{c} u=u_{w}=ax,\;v=0,\;w=0,\;T=T_{w}\text{ at }z=0,\\ u=0,\;v=0,\;T=T_{\infty }\text{ as }z\to \infty , \end{array}$$ (5)

in which *u* and *v* are the velocity components along the *x*− and *y*− directions respectively, *q*_*r*_ = −(4σ*/3k*)*∂T*^4^/*∂z* is the Rosseland radiative heat flux in which σ* is the Stefan-Boltzman constant and k* is the mean absorption coefficient (see Table 1).

**Table 1:** List of symbols.

(*x*,*y*,*z*)	Cartesian coordinate system		*Subscripts*
*u*,*v*,*w*	velocity components along the *x-*,*y*-,*z*- directions respectively	*ƒ*	fluid phase
*T*	fluid temperature	*s*	solid phase
*T*_*w*_	wall temperature	*nf*	nanofluid
*T*_*∞*_	ambient fluid temperature		
*u*_*w*_	velocity of the stretching sheet		*Greek symbols*
*f′*,*g*	dimensionless *x*- and *y*- components of velocity	*λ*	ratio of rotation rate to the stretching rate
*a*	stretching rate	*ρ*	density
*B*_*0*_	uniform magnetic field	*μ*	dynamic viscosity
*k*	thermal conductivity	*ν*	kinematic viscosity
*q*_*r*_	radiative heat flux	*α*	thermal diffusivity
*M*	Hartman number	Ω	angular velocity
*Pr*	Prandtl number	*σ*	electrical conductivity
*Rd*	radiation parameter	*η*	similarity variable
*Re*	Reynolds number	*θ*	dimensionless temperature
*q*_w_	wall heat flux	*θ*_*w*_	temperature ratio parameter
*Nu*	local Nusselt number	*ϕ*	dimensionless nanoparticle concentration
*C*_*f*_	skin friction coefficient	*τ*_*wx*,_*τ*_*wy*_	wall shear stresses along *x*− and *y*− direction

Brinkman [48] expressed the dynamic viscosity of nanofluid *μ*_*nf*_ as

$$\mu_{nf}=\frac{\mu_{f}}{{\left(1-\varphi \right)}^{2.5}},$$ (6)

the effective density *ρ*_*nf*_ and effective heat capacity (*ρc*_*p*_)_*nf*_ are expressed as [47]:

$$\rho_{nf}=(1-\phi )\rho_{f}+\phi \rho_{s},$$ (7)

$${\left(\rho c_{p}\right)}_{nf}=(1-\phi ){\left(\rho c_{p}\right)}_{f}+\phi {\left(\rho c_{p}\right)}_{s},$$ (8)

We take into account the Maxwell-Garnett model [49] for effective thermal conductivity of nanofluid *k*_*nf*_ given below:

$$\frac{k_{nf}}{k_{f}}=\;\frac{\left(k_{s}+2k_{f}\right)-2\varphi \left(k_{f}-k_{s}\right)}{\left(k_{s}+2k_{f}\right)+\varphi \left(k_{f}-k_{s}\right)}.$$ (9)

Moreover, the electrical conductivity of nanofluid *σ*_*nf*_ is given in the book by Maxwell [50] as

$$\frac{\sigma_{nf}}{\sigma_{f}}=1+\frac{3\left(\sigma_{s}-\sigma_{f}\right)\phi }{\left(\sigma_{s}+2\sigma_{f}\right)-\left(\sigma_{s}-\sigma_{f}\right)\phi }.$$ (10)

In Eqs (6)–(10), *ϕ* denotes the nanoparticle volume fraction and the subscripts *s* and *f* correspond to the solid and fluid phases respectively. Thermophysical properties of water and magnetite-Fe_3_O_4_ are given in Table 2.

**Table 2:** Thermo-physical properties of water and magnetite-Fe_3_O_4_ [10].

	*ρ*(kg/m^3^)	*C*_*p*_(J/kgK)	*K*(W/mK)	*σ*(Ω.m)^−1^
Water	997.1	4179	0.613	0.05
Fe_3_O_4_	5180	670	9.7	25000

We look for similarity solution of Eqs (1)–(4) in the following forms [5]

$$\begin{array}{c} \eta =\sqrt{\left(\frac{a}{\nu_{f}}\right)}\;z,\;u=axf^{\prime }\left(\eta \right),\;v=axg\left(\eta \right),\;w=-\sqrt{a\nu_{f}}f\left(\eta \right),\\ T=T_{\infty }+\left(T_{w}-T_{\infty }\right)\theta \left(\eta \right). \end{array}$$ (11)

In view of the above quantities, the continuity Eq (1) is identically satisfied while Eqs (1)–(5) become

$$\frac{1}{{\left(1-\phi \right)}^{2.5}\left(1-\phi +\phi \rho_{s}/\rho_{f}\right)}{{f^{\prime }}^{\prime }}^{\prime }-{f^{\prime }}^{2}+f{f^{\prime }}^{\prime }+2\lambda g-\frac{\sigma_{nf}/\sigma_{f}}{\left(1-\varphi +\varphi \rho_{s}/\rho_{f}\right)}Mf^{\prime }=0,$$ (12)

$$\frac{1}{{\left(1-\phi \right)}^{2.5}\left(1-\phi +\phi \rho_{s}/\rho_{f}\right)}{g^{\prime }}^{\prime }+fg^{\prime }-f^{\prime }g-2\lambda f^{\prime }-\frac{\sigma_{nf}/\sigma_{f}}{\left(1-\varphi +\varphi \rho_{s}/\rho_{f}\right)}Mg=0,$$ (13)

$$\frac{1}{\left(1-\phi +\phi {\left(\rho c_{p}\right)}_{s}/{\left(\rho c_{p}\right)}_{f}\right)}\frac{1}{\text{Pr}}{\left(\left(k_{nf}/k_{f}+Rd{\left(1+\left(\theta_{w}-1\right)\theta \right)}^{3}\right)\theta^{\prime }\right)}^{\prime }+f\theta^{\prime }=0,$$ (14)

$$\begin{array}{c} f\left(0\right)=0,\;g\left(0\right)=0,\;f^{\prime }\left(0\right)=1,\;\theta \left(0\right)=1,\\ f^{\prime }\left(\infty \right)=0,\;g\left(\infty \right)=0,\;\theta \left(\infty \right)=0, \end{array}$$ (15)

in which Pr = (*μc*_*p*_)_*f*_ /*k*_*f*_ is the Prandtl number of the base fluid, *Rd* = 16σ*T_*∞*_^3^/3k*k_*f*_ denotes the radiation parameter, $M=\sigma B_{0}^{2}/\rho_{f}\Omega$ is the magnetic field parameter and *λ* = Ω/*a* is the ratio of rotation rate to the stretching rate. The quantities of practical interest are the skin friction coefficients *C*_*fx*_, *C*_*fy*_ and the local Nusselt number *Nu*_*x*_ defined as follows.

$$C_{fx}=\frac{\tau_{wx}}{\rho_{f}u_{w}^{2}},\;C_{fy}=\frac{\tau_{wy}}{\rho_{f}u_{w}^{2}},\;Nu_{x}=\frac{xq_{w}}{k_{f}(T_{w}-T_{\infty })},\;$$ (16)

where $\tau_{wx}={\tau_{zx}|}_{z=0}$ and $\tau_{wy}={\tau_{zy}|}_{z=0}$ are the wall shear stresses and *q*_*w*_ is the wall heat flux given by

$$\tau_{wx}=\mu_{nf}{\frac{\partial u}{\partial z}|}_{z=0},\;\tau_{wy}=\mu_{nf}{\frac{\partial v}{\partial z}|}_{z=0},\;q_{w}=-k_{nf}{{\frac{\partial T}{\partial z}|}_{z=0}+q_{r}|}_{z=0}.$$ (17)

Using Eq (11) in Eq (16) one obtains

$$\sqrt{Re_{x}}C_{fx}=\frac{1}{{\left(1-\phi \right)}^{2.5}}f^{\prime \prime }(0),\;\sqrt{Re_{x}}C_{fy}=\frac{1}{{\left(1-\phi \right)}^{2.5}}g^{\prime }(0),\;\frac{Nu_{x}}{\sqrt{Re_{x}}}=-\left(\frac{k_{nf}}{k_{f}}+Rd\theta_{w}^{3}\right)\theta^{\prime }(0),$$ (18)

in which *Re*_*x*_ = *u*_*w*_*x*/*v*_*f*_ is the local Reynolds number.

In the present article, we employed the Tiwari and Das model [47] to address the rotating flow and heat transfer of Fe_3_O_4_-water ferrofluid driven by a linearly stretching surface. Numerical solutions of the Eqs (12)–(14) subject to the conditions Eq (15) are computed by using shooting method with Runge-Kutta fifth order integration scheme. In all the calculations the volume fraction is considered in the range 0 ≤ *ϕ* ≤ 0.2 (as *ϕ* beyond 0.2 is not physically realizable due to accumulation) while the Prandtl number Pr = 6.2 for water is used. We have compared our results with available studies of Wang [1] and Nazar et al. [3] in the case of pure fluid and found very good agreement (see Table 3).

**Table 3:** Comparison of current result with previous studies for special cases (*ϕ* = 0,*M* = 0).

*λ*	Wang [1]		Nazar et al. [3]		Present	
	*f″*(0)	*g*′(0)	*f″*(0)	*g*′(0)	*f″*(0)	*g*′(0)
0	-1	0	-1	0	-1	0
0.5	-1.1384	-0.5128	-1.1384	-0.5128	-1.13838	-0.51276
1.0	-1.3250	-0.8371	-1.3250	-0.8371	-1.32503	-0.837089
2.0	-1.6523	-1.2873	-1.6523	-1.2873	-1.65235	-1.28726

## Numerical Results and Discussion

The numerical values of local Nusselt number $Nu_{x}/\sqrt{{\text{Re}}_{x}}$ for some values of parameters are computed in Table 4. The magnitude of $Nu_{x}/\sqrt{{\text{Re}}_{x}}$ grows sharply with the volume fraction *ϕ* is when larger wall to ambient temperature ratio is accounted. Moreover *$Nu_{x}/\sqrt{{\text{Re}}_{x}}$* has direct as well as non-linearly relationship with the radiation parameter *Rd*. This outcome is true for both linear and non-linear radiation cases.

**Table 4:** Values of local Nusselt number $Nu_{x}/\sqrt{{\text{Re}}_{x}}$ for different values of embedded parameters.

				*Rd = 1*		
*ϕ*	*M*	*λ*	*Rd = 0*	Linear radiation	Nonlinear radiation	
					*θ*_*w*_ = 1.1	*θ*_*w*_ = 1.5
0.1	1	0	1.88862	2.29587	2.36871	2.70039
		0.5	1.85750	2.23679	2.30471	2.60866
		1	1.78831	2.10820	2.16559	2.41046
		2	1.62561	1.82975	1.86624	2.00376
0.1	0	1	1.83496	2.16281	2.22182	2.46987
	0.5		1.81436	2.14190	2.20074	2.45110
	1		1.78831	2.10820	2.16559	2.41046
	2		1.72931	2.02393	2.07670	2.30010
0	1	0.5	1.66171	2.15308	2.23565	2.59822
0.05			1.75604	2.18830	2.26323	2.59451
0.1			1.85750	2.23679	2.30471	2.60866
0.2			2.09314	2.38075	2.43596	2.68996

**Fig 2:**
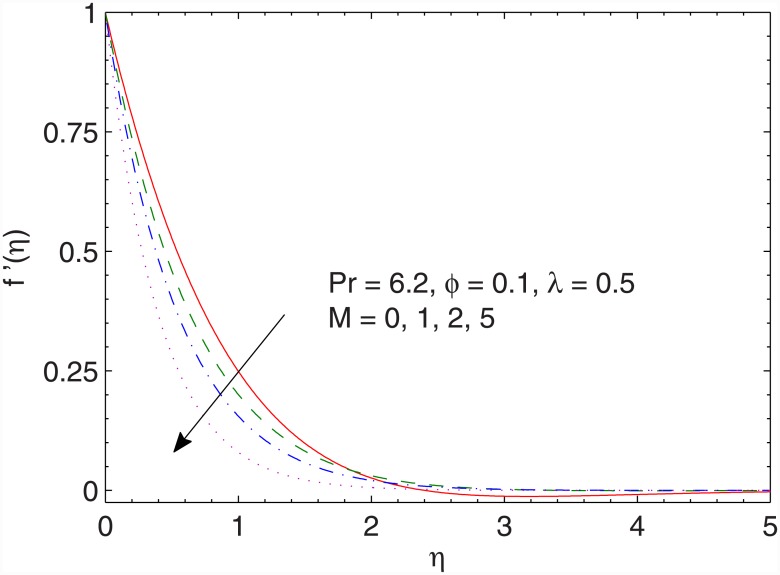
Effect of *M* on *f*′(*η*).

**Fig 3:**
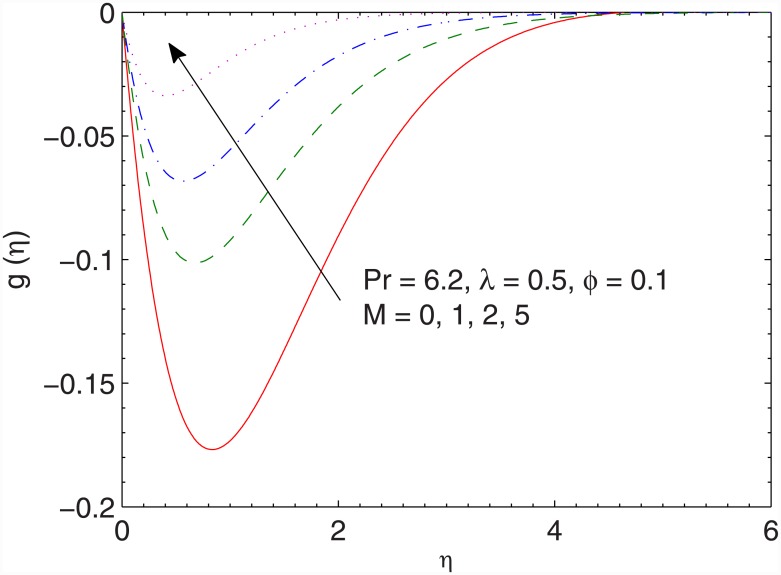
Effect of *M* on *g*(*η*).

Figs 2 and 3 preserve the effects of magnetic field strength on the dimensionless *x*− and *y*− components of velocity respectively. The function *g*(*η*) has a parabolic profile and its value is negative revealing that flow occurs in the negative y− direction. As the Hartman number *M* enlarges, the flow decelerates in both *x*− and y− directions. Physically, the presence of magnetic field restricts the fluid motion due to which a thinner boundary layer appears for stronger magnetic field strength.

**Fig 4:**
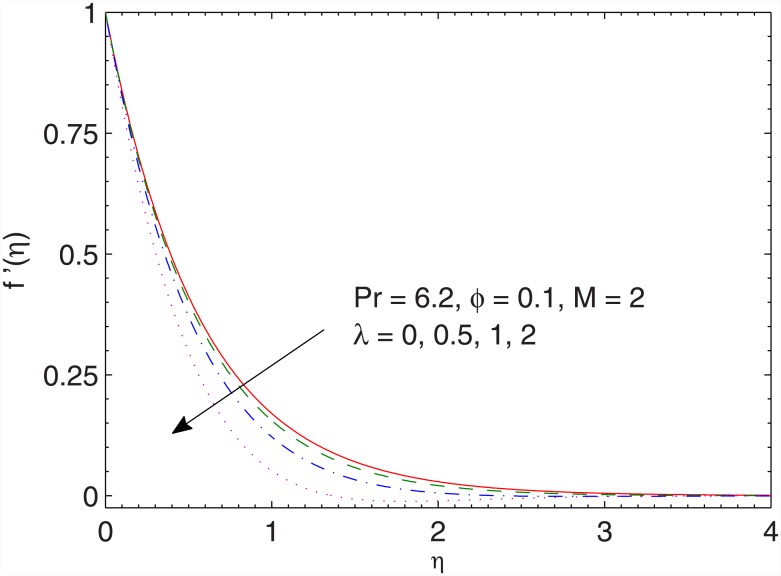
Effect of *λ* on *f*′(*η*).

**Fig 5:**
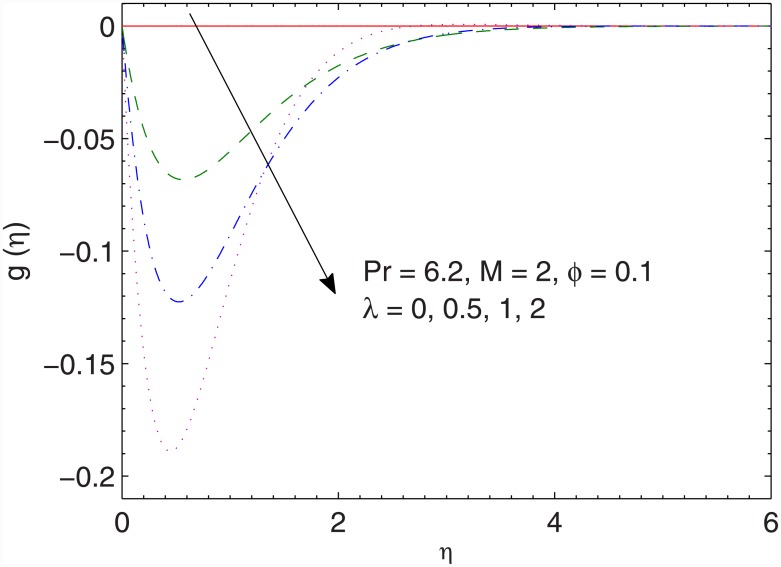
Effect of *λ* on *g*(*η*).

In Figs 4 and 5, we portray the effects of ratio *λ* on the *x*− and *y*− components of velocity respectively. Larger values of *λ* indicates smaller stretching rates (along the *x*− direction) compared to the rotation rate. Due to this fact, the *x*− component of velocity *f*′(*η*) is inversely proportional to *λ* and magnitude of the y− component of velocity *g*(*η*) increases when *λ* is increased.

**Fig 6:**
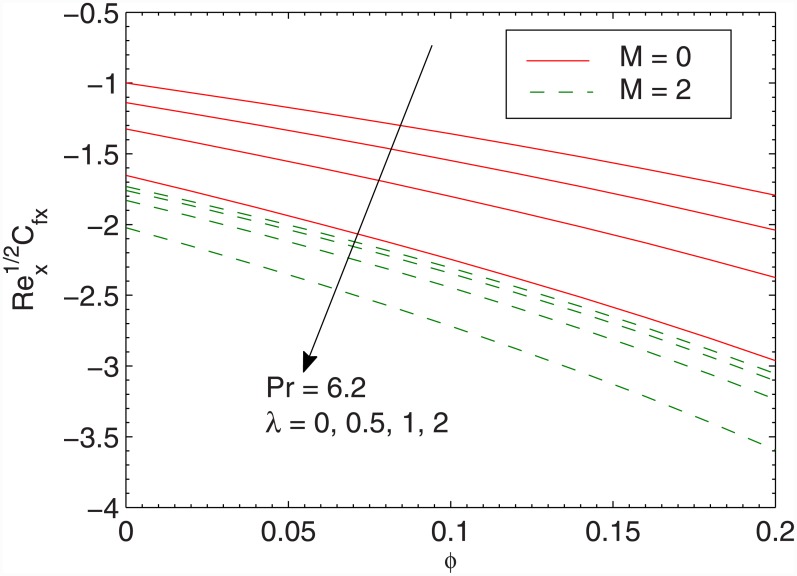
Effect of *λ*, *M* and *ϕ* on $\sqrt{{\text{Re}}_{x}}C_{fx}$.

**Fig 7:**
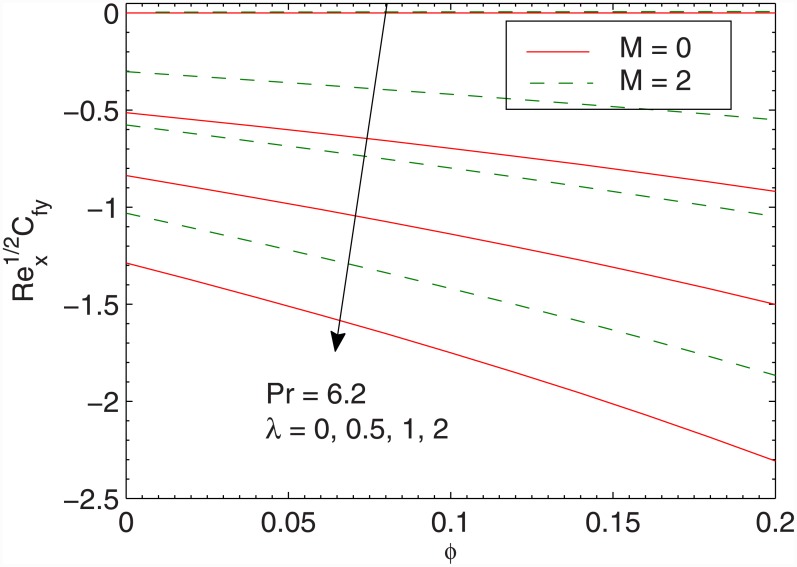
Effect of *λ*, *M* and *ϕ* on $\sqrt{{\text{Re}}_{x}}C_{fy}$.

In Figs 6 and 7 we plot the skin friction coefficients $Re_{x}{}^{1/2}{\text{ C}}_{fx}$ and $Re_{y}{}^{1/2}{\text{ C}}_{fy}$ against the volume fraction *ϕ* for different values of *λ*. The values of skin friction coefficients $Re_{x}{}^{1/2}{\text{ C}}_{fx}$ and $Re_{y}{}^{1/2}{\text{ C}}_{fy}$ are negative since the fluid applies stress on the stretching wall (that causes the flow). Clearly the magnitudes of $Re_{x}{}^{1/2}{\text{ C}}_{fx}$ and $Re_{y}{}^{1/2}{\text{ C}}_{fy}$ are decreasing functions of *M*. This is attributed to the fact that transverse magnetic field has a tendency to create a drag (known as Lorentz force that resists the transport phenomenon). This leads to the deceleration of flow and enhancement in the surface shear stress. We also observe that wall shear stresses increase nonlinearly with an increase in *ϕ*. This means that larger force will be required to displace the fluid over the sheet when larger concentration of *Fe*_3_*O*_4_ particles in water is considered. Physically, an increase in *ϕ* enhances the viscous force which induces larger stress at the wall (since *τ*_*yx*_ = μ_*nf*_∂*u*/*∂y*) and consequently the skin friction coefficient enlarges. The outcome is similar for both magnetic and non-magnetic nanoparticles. We also conclude that tangential stress *τ*_*zx*_ is more sensitive to parameter *λ* than the tangential stress *τ*_*zy*_.

**Fig 8:**
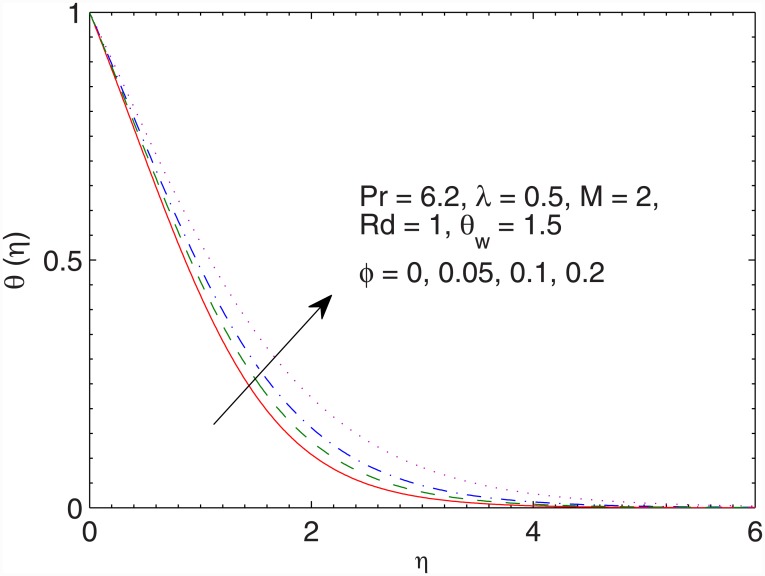
Effect of *ϕ* on *θ*(*η*).

The influence of volume fraction *ϕ* on the temperature *θ* is sketched in the Fig 8. As expected, the intense viscous force due to the consideration of large *ϕ* develops thicker thermal boundary. It is also noted that wall temperature gradient augments with an increase in *ϕ*.

**Fig 9:**
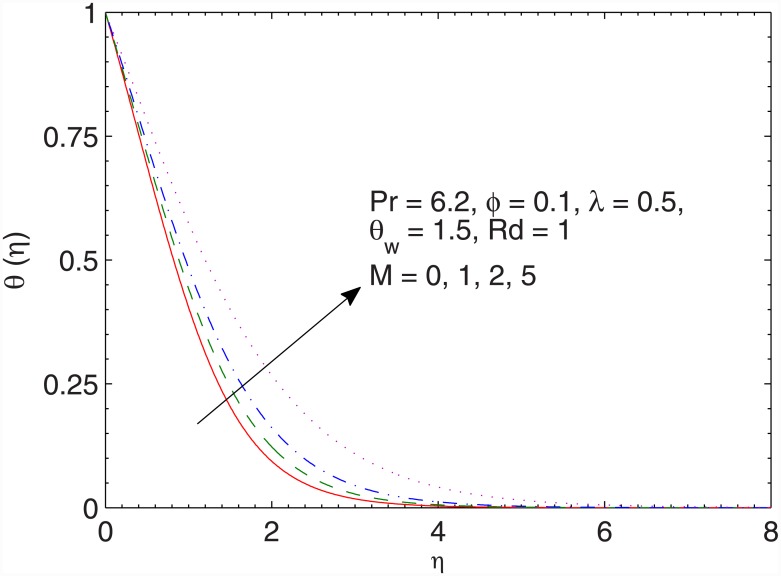
Effect of *M* on *θ*(*η*).

**Fig 10:**
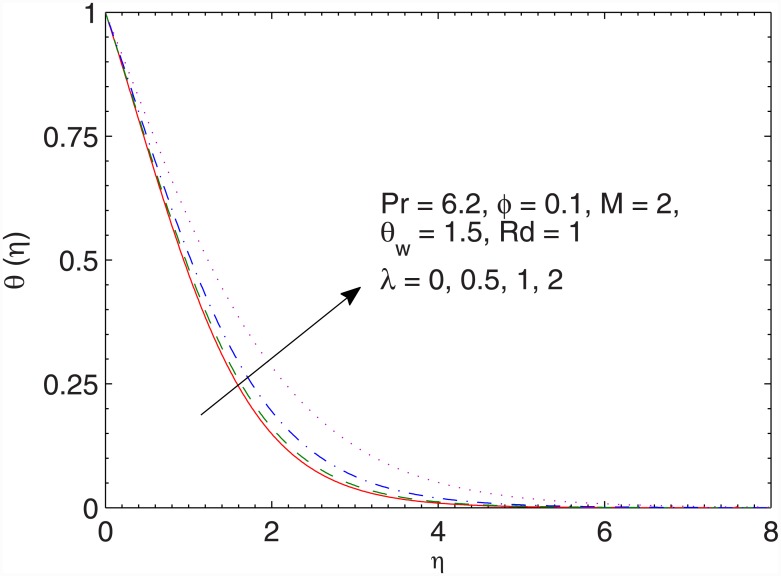
Effect of *λ* on *θ*(*η*).

Fig 9 contains the impact of magnetic field on the temperature *θ*. The resistance associated with the Lorentz force due to the applied magnetic field enhances the temperature. Due to this reason, temperature rises and thermal boundary layer gets thicker when *M* is incremented. The behavior of ratio *λ* on thermal boundary layer is qualitatively similar to that of *M* (see Fig 10). However the effects are prominently felt when *λ* is varied.

**Fig 11:**
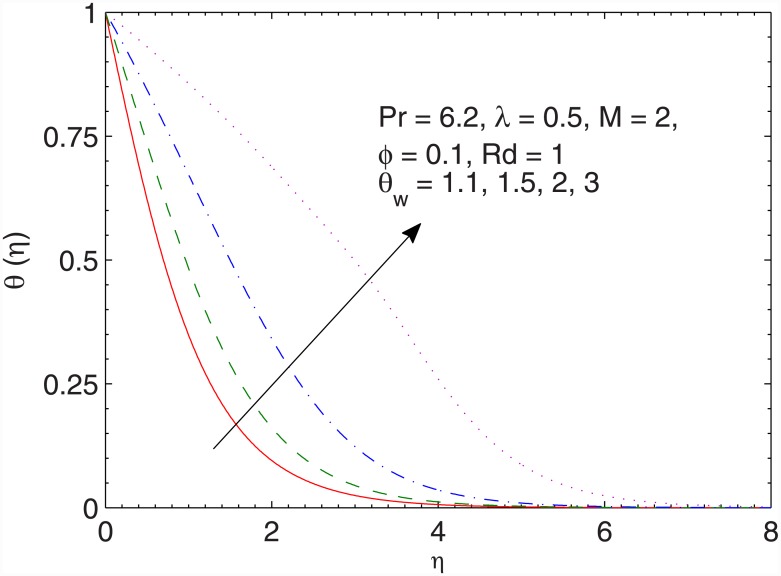
Effect of *θ*_*w*_ on *θ*(*η*).

Temperature profiles for several values of temperature ratio parameter *θ*_*w*_ are sketched in Fig 11. Unlike the linear radiation case, the profiles change from normal shape to the S-shaped thicker profiles when *θ*_*w*_ is increased. Here the thermal boundary layer thickness is controlled by the effective thermal diffusivity *α*_*eff*_ = *α* + 16σ*T^*3*^/3*ρC*_*p*_k*) which is a function of temperature. Since the sheet is hotter than the fluid therefore one expects *a*_*eff*_ to be larger near the sheet than at the ambient fluid. Due to this reason, an increase in wall to ambient temperature ratio parameter *θ*_*w*_ tends to a decrease the temperature gradient near the surface which results in the point of inflection. More precisely, the concavity of the temperature function changes in [0,*∞*) when *θ*_*w*_ is sufficiently large. On the other hand, the concavity has been preserved in case of linear radiation heat transfer. As *θ*_*w*_ enlarges, this corresponds to larger temperature difference between wall and the ambient which eventually imparts a thicker thermal boundary layer. The results are in accordance with the previously published articles.

**Fig 12:**
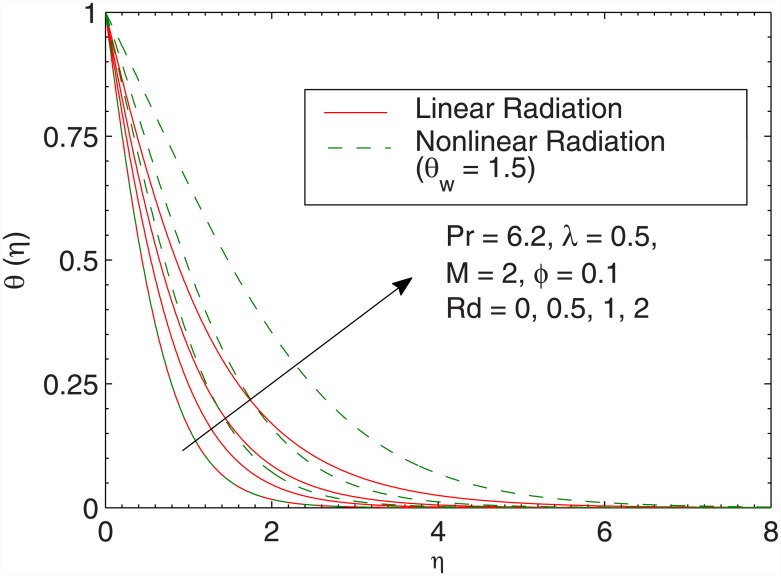
Effect of *Rd* on *θ*(*η*).

**Fig 13:**
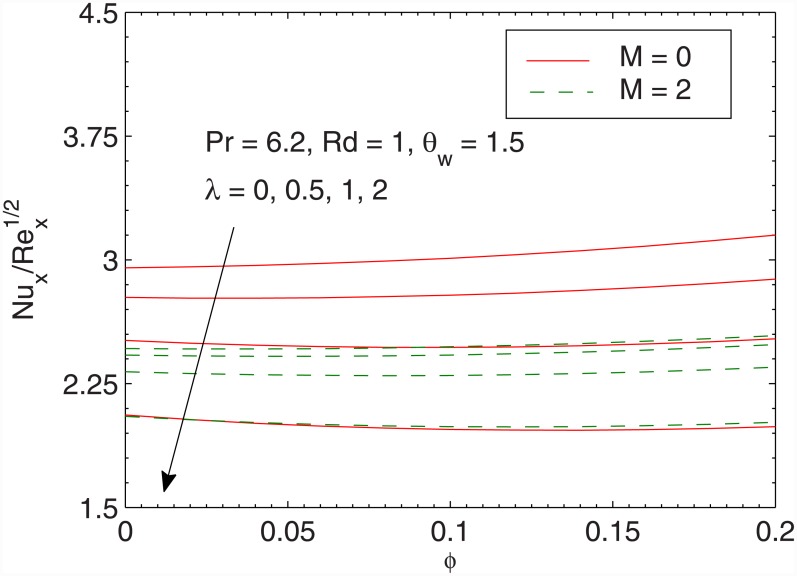
Effects of *λ*, *M* and *ϕ* on $Nu_{x}/\sqrt{{\text{Re}}_{x}}$.

**Fig 14:**
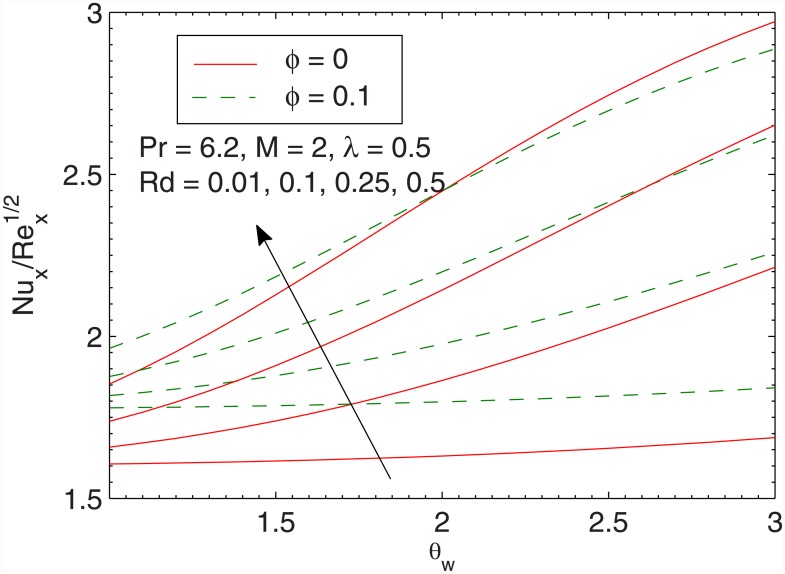
Effects of *Rd* and *θ*_*w*_ on $Nu_{x}/\sqrt{{\text{Re}}_{x}}$.

Fig 12 gives the effect of radiation parameter *Rd* in both the cases of linear and non-linear radiative heat fluxes. In accordance with Cortell [46], wall slope of temperature tends to a constant finite value when radiation parameter *Rd* is increased for linear radiation case. Such effect is not preserved in the case of the nonlinear radiation. Temperature *θ* appears to be larger when larger radiation parameter is accounted. Moreover, temperature *θ* rises sharply in non-linear radiation when compared with the linear radiation. From Fig 12 we also envisage that linear and non-linear radiation results are identical only when *Rd* is small and *θ*_*w*_ is close to one. The difference between linear and non-linear radiation continues to grow as the radiation parameter is gradually increased, a fact that is also found in [45]. Fig 13 shows that local Nusselt number $Nu_{x}/\sqrt{{\text{Re}}_{x}}$ has direct relationship with the volume fraction *ϕ* is increased. Notably, the heat transfer rate from the sheet significantly varies only for small values of *λ*. Fig 14 plots the local Nusselt number $Nu_{x}/\sqrt{{\text{Re}}_{x}}$ against *θ*_*w*_ for various values of *Rd*. We observe a sharp growth in heat transfer rate when *θ*_*w*_ is increased. The slope of this function further increases when *Rd* is increased. Here the comparison of results for pure water and ferrofluid is also given.

## Concluding Remarks

Revolving flow and heat transfer of magnetite-water ferrofluid over a deformable surface is explored through Tiwari and Das model. The developed non-linear boundary value problem is tackled by a numerical approach. The major aspects of this work are highlighted below:

1. The *y*− component of velocity is negative and has a parabolic distribution.
2. The parameter *λ*, which gives the ratio of rotation rate to the stretching rate, has opposite effects on the *x*− and *y*− components of velocity qualitatively.
3. For sufficiently large wall to ambient temperature ratio, temperature function *θ* has an interesting S-shaped profile which indicates the existence of adiabatic case.
4. Variations in *x*− and *y*− components of velocity with the nanoparticle volume fraction *ϕ* is non-monotonic.
5. The velocity distributions *f′* and *g* decrease when larger values of magnetic field parameter *M* are employed.
6. Skin friction coefficient and local Nusselt number are directly proportional to the nanoparticle volume fraction *ϕ*.
7. Local Nusselt number attenuates when magnetic field strength is intensified.
